# Anti-Amyloid-β-Mediated Positron Emission Tomography Imaging in Alzheimer's Disease Mouse Brains

**DOI:** 10.1371/journal.pone.0051958

**Published:** 2012-12-21

**Authors:** Daniel McLean, Michael J. Cooke, Yuanfei Wang, David Green, Paul E. Fraser, Peter St George-Hyslop, Molly S. Shoichet

**Affiliations:** 1 Department of Chemical Engineering and Applied Chemistry, University of Toronto, Toronto, Ontario, Canada; 2 Institute of Biomaterials and Biomedical Engineering, University of Toronto, Toronto, Ontario, Canada; 3 Center for Spatio and Temporal Targeting and Amplification of Radiation Responses, Princess Margaret Hospital, Toronto, Ontario, Canada; 4 Tanz Centre for Research in Neurodegenerative Diseases, Departments of Medicine (Neurology) and Medical Biophysics, University of Toronto, Toronto, Ontario, Canada; 5 Cambridge Institute for Medical Research, University of Cambridge, Cambridge, United Kingdom; 6 Department of Chemistry, University of Toronto, Toronto, Ontario, Canada; National University of Ireland - Galway, Ireland

## Abstract

Antibody-mediated imaging of amyloid β (Aβ) in Alzheimer's disease (AD) offers a promising strategy to detect and monitor specific Aβ species, such as oligomers, that have important pathological and therapeutic relevance. The major current limitation of antibodies as a diagnostic and imaging device is poor blood-brain-barrier permeability. A classical anti-Aβ antibody, 6E10, is modified with 10 kDa polyethylene glycol (PEG) and a positron emitting isotope, Copper-64 (t_½_ = 12.7 h), and intravenously delivered to the TgCRND8 mouse model of Alzheimer's disease. Modification of 6E10 with PEG (6E10-PEG) increases accumulation of 6E10 in brain tissue in both TgCRND8 and wild type control animals. 6E10-PEG differentiates TgCRND8 animals from wild type controls using positron emission tomography (PET) and provides a framework for using antibodies to detect pathology using non-invasive medical imaging techniques.

## Introduction

Alzheimer's disease (AD) is a class of dementia that results in neurodegeneration, memory loss and impaired cognition and is characterized by the accumulation of senile plaques (aggregated amyloid (Aβ) protein) and neurofibrillary tangles (hyperphosphorylated tau protein) in the brain [1], [2]. AD affects over 24 million individuals across the globe and prevalence is expected to double over the next 30 years in Western countries due to a rapidly ageing population [3], [4]. Internationally, the prevalence of dementia is expected to increase by a factor of three, which is creating a significant social and economic burden globally [4].

Effective early diagnosis and monitoring of AD remains elusive as most patients are diagnosed through observation of impaired cognition and memory; approximately 50% of cases are estimated to go undiagnosed at early-stages [5]. Two medical imaging techniques, structural magnetic resonance imaging (which measures regional tissue loss in the brain) and fluorodeoxyglucose positron emission tomography (which measures changes in metabolic activity), have been implemented in the clinic with some success [6], [7]. Functional and structural techniques have inherent limitations because they measure the loss of neuronal activity rather than the causative pathology [8]. Small molecule imaging ligands that target Aβ plaques, such as Pittsburgh Compound B (PIB) which targets compact Aβ plaques, are examples of molecular imaging tools that are showing success in detecting Aβ in AD patients when labelled with an appropriate positron emitting isotope [9]. The primary advantage of many small molecules, such as PIB, is their ability to cross the blood-brain barrier (BBB) when administered systemically; however, it is particularly difficult to design small molecules to target a specific, pathologically relevant isoform of Aβ [10].

Anti-Aβ antibodies are interesting imaging ligands because they address the key specificity disadvantage associated with using small molecules, yet anti-Aβ antibodies have limited ability to cross the BBB [11]. One approach to improve BBB penetration of antibodies is to mechanically disrupt the tight junctions using either a hyperosmotic sugar solution delivered locally through the carotid artery [12] or through image-guided focused ultrasound [13]; however, these approaches are inherently limited by their invasiveness and also open the BBB to all molecules in circulation. Alternatively, antibodies can be chemically modified with a ligand that enables receptor mediated transport across the BBB, such as coupling to an anti-insulin receptor antibody [14]. Transport across the BBB can also be enhanced by altering the surface charge of the antibody, such as coupling it to positively charged polyamines [15].

A widely employed strategy to improve BBB penetration of macromolecules is to mimic the transport of cholesterol vesicles across the BBB because these vesicles are large and thus similar in size to macromolecules or nanoparticles. Cholesterol vesicles are decorated with lipoproteins that enable receptor-mediated transport across the BBB. Chemical immobilization of apolipoprotein E on nanoparticles has been shown to increase transport of nanoparticles into the brain [16]. Further, many polymers such as poly(*n*-butyl cyanoacrylate) [17] or PEG [18] have similar secondary structures to lipoproteins and can act as ligands for the receptors that bind lipoproteins (such as low-density lipoprotein receptors). PEG is particularly attractive for this purpose because it is widely regarded as biocompatible [19] and is available commercially in a variety of molecular weights and with a variety of reactive functional groups to enable tethering to an antibody.

When designing a diagnostic device for use in AD, a disease that takes many years to progress, it is essential to use minimally-invasive approaches such as systemic delivery of a chemically-modified antibody. Previously, the classical anti-amyloid β antibody, 6E10, was shown to stain amyloid β plaques after intracerebral injection in 8 month old TgCRND8 mice, while wild type control mice showed no binding or retention of the antibody [20]. Notwithstanding these important results, this study is not translatable to the clinic because: (1) intracerebral injections are invasive, requiring a craniotomy and insertion of the needle which disturbs cortical tissue; and (2) brains were examined using standard histological techniques that required brain tissue to be harvested. To address these issues, antibody-mediated brain imaging of amyloid β was investigated in terms of improved BBB penetrability after systemic administration and non-invasive imaging techniques [21].

**Figure 1:**
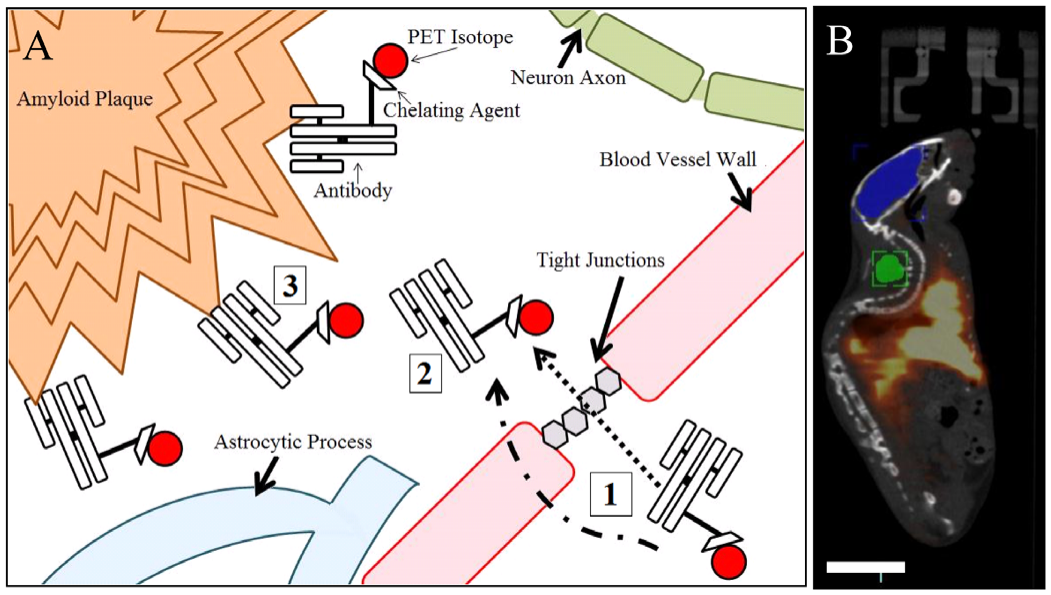
Antibody modification with a Copper chelator and positron emitter ^64^Cu allows quantification of contrast agent accumulation in the brain using Positron Emission Tomography. (A) A schematic of the imaging strategy design highlighting the antibody, copper chelator (1,4,7,10-Tetraazacyclododecane-1,4,7,10-tetraacetic acid mono (N-hydroxysuccinimide ester), DOTA-NHS-ester) and positron emitter (^64^Cu). Antibodies are delivered intravenously (1). Antibodies penetrate into brain tissue through intracellular and paracellular transport mechanisms and diffuse in brain tissue where they bind to Aβ (2). Antibodies are retained in the brain through binding to Aβ plaques (3). (B) Fusing the PET image with a CT image allows anatomical features to serve as a reference when drawing regions of interest. An example region of interest drawn in the brain is shown in yellow, while reference skin and muscle tissue is shown in the green region of interest. The entire brain was quantified in all experiments. Scale bar is 1.5 cm.

In this study, we show that 6E10 can be used for non-invasive differentiation of amyloid-β bearing TgCRND8 mice (a robust model of Alzheimer's disease) from wild type littermates. 6E10 was radiolabelled with ^64^Cu (t_1/2 = _12.7 h), a positron emitting isotope, via an intermediate chelating agent (1,4,7,10-Tetraazacyclododecane-1,4,7,10-tetraacetic acid or commonly known as DOTA [22]) so that the penetration of the antibody across the BBB and binding to Aβ plaques could be traced using PET imaging (Figure 1A). To enhance BBB permeability, we modified 6E10 with PEG because previous studies with nanoparticles have shown improved BBB permeability after PEG-modification [18]. After intravenous injection, we observed improved penetration of PEG-modified 6E10 (6E10-PEG) and non-invasive differentiation of TgCRND8 mice from wild type controls likely through binding Aβ. We also measured concentrations of 6E10 in the TgCRND8 and wild type control brains after hyperosmotic disruption of the BBB that provides further evidence of pathological detection. We successfully used 6E10 to discriminate between TgCRND8 and wild type mice. These results demonstrate an innovation in the use of antibodies for the detection of Aβ pathology using live-animal imaging that will enable detection of specific types of Aβ pathology in the future.

## Materials and Methods

### 2.1 Animals

Animal experiments were carried out in accordance with the guidelines provided by the Canadian Council for Animal Care and reviewed by in-house research ethics approval boards at the University of Toronto and the University Health Network. TgCRND8 mice were maintained on a 129 background. The TgCRND8 mouse was used as it is widely accepted as a robust model of Aβ deposition [23]. Mice carrying the Swedish and Indiana mutations were differentiated from wild type (WT) mice by RT-PCR analysis of tail DNA.

### 2.2 Radiolabelling Antibodies

The labelling of covalent modification of 6E10 with DOTA and 10 kDa PEG is described in supplementary information S1. 6E10-DOTA or 6E10-PEG-DOTA conjugates were labelled with the positron emitting isotope ^64^Cu. ^64^Cu was produced by the Centre d'imagerie moleculaire de Sherbrooke (CIMS, Sherbrooke, QC) the day prior to radiolabelling. 1 mg of 6E10-DOTA was incubated with 5 mCi of ^64^Cu in PBS at room temperature for 2 h. A small amount of the unpurified mixture was placed on an instant thin layer chromatography (iTLC) sheet (iTLC-SG, Pall) and resolved in 0.1 M, pH5 citrate buffer. ITLC sheets were visualized with a Cyclone Plus Phosphor Imager device (Perkin Elmer) to verify chelation. 6E10-^64^Cu mixtures were purified from unchelated ^64^Cu using an Amicon Ultra-0.5 mL centrifugal 30 kDa molecular weight cut-off filter unit (UFC5030, Millipore). Mixtures were rinsed 4 times with saline and purity confirmed using ITLC. Only mixtures of greater than 95% purity were used for *in vivo* experiments. All animals were injected within 12 h of synthesis.

### 2.3 Intravenous Injection of 6E10-^64^Cu and 6E10-PEG-^64^Cu

12 month old animals were used because previous experiments indicate that 6E10 only binds to plaques in animals over 8 months of age without antigen retrieval [20]. 12 month old TgCRND8 and wild type littermate controls were given intravenous tail vein injections of 100 µg of 6E10-^64^Cu (TgCRND8 n = 3, WT n = 3) or 6E10-PEG-^64^Cu ^64^Cu (TgCRND8 n = 3, WT n = 3). The amount of ^64^Cu in the administration syringe was measured using a dose calibrator (CRC-15R, Capintec) before and after injection in order to determine the precise dose of ^64^Cu administered to each animal. Animals received a minimum of 50 µCi. Animals were immediately anaesthetized with isoflurane (5% induction, 2% maintenance) and placed on a PET imaging bed (Minnerve). All imaging was conducted at the Spatio-Temporal Targeting and Amplification of Radiation Response (STTARR) Innovation Centre (University Health Network, Toronto, ON) Animals were imaged approximately 5 min after injection for 15 min using a Siemens Micro PET Focus 220, this time point is referred to using the median time, 10 min. Animals were then transferred to a GE Locus Ultra microCT for a 15 s computed tomography scan to provide spatial information. Animals were re-imaged with the microPET and microCT at 2 h, 4 h, and 12 h. Animals were not kept under anaesthesia in between time points. At 12 h, animals were sacrificed by cardiac puncture and all organs were collected and dose of ^64^Cu measured in a gamma counter (Wallac Wizard 3, Perkin Elmer). A standard curve of ^64^Cu was prepared (cross-calibrated to the dose calibrator) in order to quantify the percent injected dose per gram of tissue for each organ.

### 2.4 Hyperosmotic Disruption of the Blood-Brain Barrier

10 month old TgCRND8 (n = 4) and wild type littermate controls (n = 4) were given intracarotid arterial injections of 1.8 M arabinose (Sigma-Aldrich) to temporarily open the blood-brain barrier. 10 month old TgCRND8 animals contain comparable amounts of Aβ plaques to the 12 month old animals used previously. Surgical protocol was modelled after previously described techniques [12]. Briefly, animals were anaesthetized with isoflurane, an incision was made in the neck and the internal carotid artery was dissected and sutured in 2 locations. The blood flow was temporarily stopped while an incision was made in the artery and a catheter (PE-8 tubing, SAI Infusion) inserted into the artery. The catheter was sutured into place and the distal suture removed. 75 µL of 1.8 M arabinose was infused over 1 min followed by 75 µL of 6E10-^64^Cu over 1 min and to ensure all 6E10 was out of the needle and catheter 50 µL of saline over 1 min. Animals received a minimum of 50 µCi. The neck incision was sutured and the animals placed on a PET imaging bed. Animals were dynamically imaged under anaesthesia for 4 h, followed by a static CT scan and sacrificed as described in Section 2.4.

### 2.5 Reconstruction, Fusion and Quantitation of PET and CT Images

Static PET scans from the intravenous studies were reconstructed as a single frame with a 128×128×95 matrix (voxel size 1.9×1.9×0.8 mm) using an iterative reconstruction method (OSEM3D/MAP, 2 subsets of 9 iterations). Dynamic PET scans from the intracarotid arterial studies were reconstructed as twenty-four 10 min frames using the same algorithm as described above. CT scans were reconstructed using with a 256×256×680 image matrix (voxel size 154×154×154 µm). PET and CT data sets were manually fused using Inveon Research Workplace (Siemens). Intensity on the PET images was increased to reveal the animals shape and allow the head, body, arms and legs to be matched to the CT image. The CT images were used to provide spatial/structural information for locations of the brain in the PET images. A region of interest was drawn in the brain and the amount of ^64^Cu quantitated as a mean percent injected dose per gram of tissue (%ID/g) at each time point. In the intravenous studies a reference region was drawn in the neck muscle for comparison (Figure 1B). Quantitation of these small volumes was possible because the skull provided important spatial information that allowed the brain to be clearly differentiated from other tissues. Each image was fused and quantified three times to reduce bias due to fusing error. Radioactive decay was corrected for in all %ID/g-tissue measurements reported using a standard exponential decay formula.

### 2.6 Biodistribution of Rabbit IgG and PEG-modified Rabbit IgG

Five month old wild type 129 mice were injected with 1 mg of rabbit IgG (Sigma-Aldrich). Younger animals were employed in this wild type control experiment because there was no need to wait for Aβ pathology to develop. Rabbit IgG was also modified with 10 kDa PEG as described above and administered by tail vein injection. Rabbit IgG and PEG-IgG was allowed to circulate for 6, 18, 48 h (n = 4 for each time point and antibody). Animals were sacrificed by cardiac puncture and the blood and brains collected. Heparinised (Sigma-Aldrich) blood was centrifuged to remove red blood cells and the serum fraction collected. Brains were homogenized in PBS, centrifuged and the supernatant collected. A rabbit IgG ELISA was developed using a sheep anti-rabbit-IgG capture antibody (Sigma-Aldrich), a goat anti-rabbit-IgG-alkaline phosphatase detection antibody (Sigma-Aldrich) and developed with a pNPP solution (Sigma-Aldrich).

### 2.7 Statistical Analysis

All data was analyzed using either 1-, 2- or 3-factor analysis of variance (ANOVA) followed by the appropriate post-hoc test to determine individual differences (SigmaPlot). Error bars represent standard deviation and significances are indicated at the p = 0.05 level.

## Results

### 3.1 PET Imaging of Intravenous Delivered 6E10 and 6E10-PEG in TgCRND8 vs. Wild Type Mice

**Figure 2:**
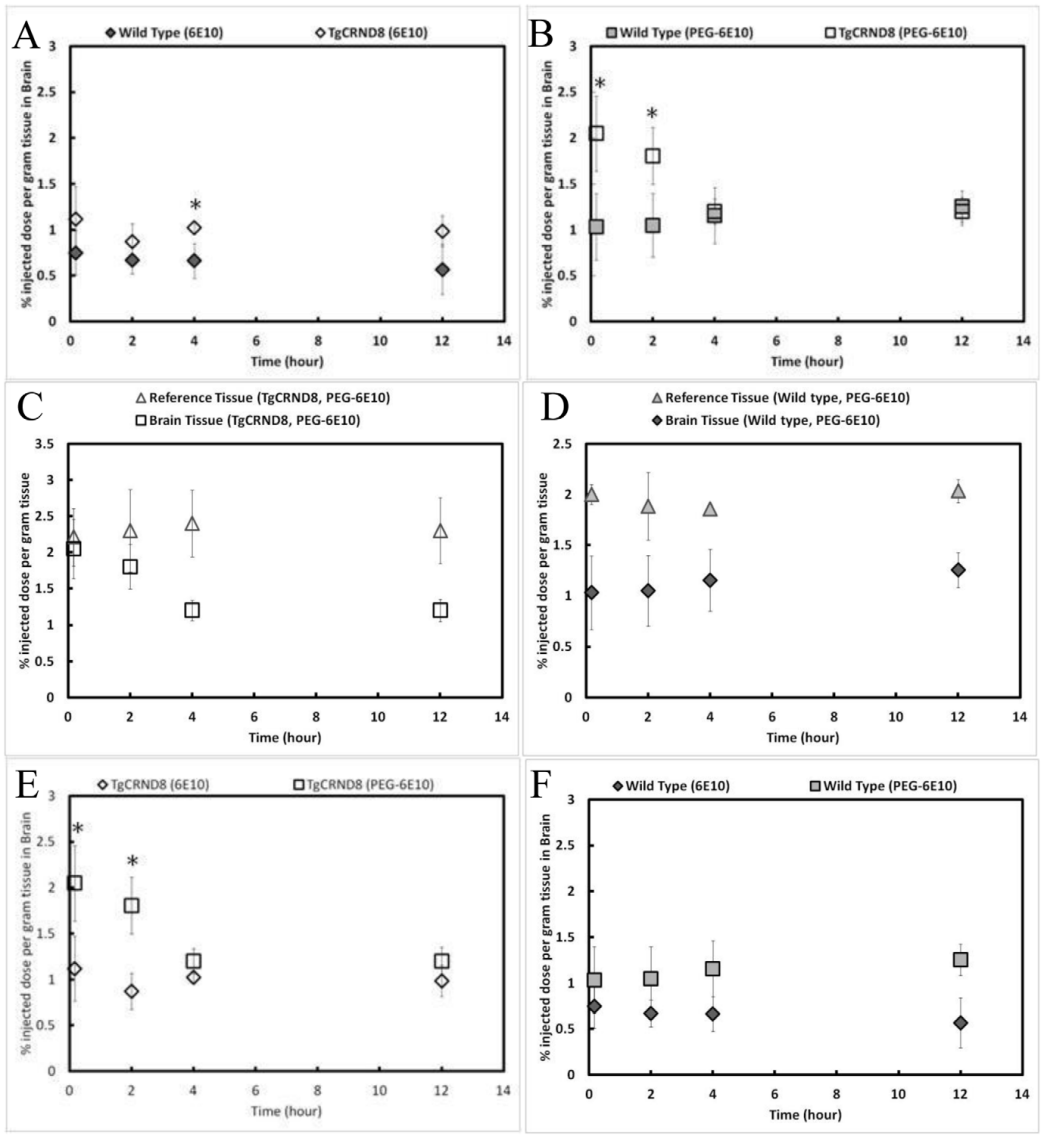
Systemic Delivery of Anti-amyloid-β antibody, 6E10, binds differently in brain tissue of amyloid-β expressing TgCRND8 mice vs. that of native wild type mice. (A) Percentage injected dose per gram of tissue, as quantified by PET, differentiates between TgCRND8 (unfilled shapes) and wild type mice (filled shapes) at 4 h (p = 0.041) using 6E10-^64^Cu(diamonds). (B) PEG-modification of 6E10-^64^Cu (squares) increases brain penetration and can distinguish between TgCRND8 and wild type mice at 10 min (p = 0.001) and 2 h (p = 0.009). (C) PET signal in the brain tissue of TgCRND8 mice decreases over time, whereas the reference tissue (triangles) stays constant, after injection with PEG-modified 6E10. (D) PET signal from the brain and reference tissue in wild type mice stays constant over 12 h. (E) PEG-modified 6E10, vs. 6E10, significantly (p = 0.003) increases permeation into brain tissue in TgCRND8 animals at 10 min and 2 h. Post-hoc Holm-Sidak test revealed significant differences at 10 min (p = 0.003) and 2 h (p = 0.002). (F) PEG-modified 6E10 similarly increases permeation of antibody into brain tissue in wild type controls (p<0.001). Post-hoc Holm-Sidak test revealed no significant differences at any time point. For all graphs, n = 3, mean ± standard deviation plotted.

In order to non-invasively measure the amount of 6E10 antibody that would accumulate in the brain of transgenic TgCRND8 Alzheimer's disease mice vs. wild type mice, 6E10 was modified with both DOTA, to chelate ^64^Cu, and PEG, to enhance penetration across the blood-brain barrier (BBB, see Figure S1 and Table S1 for characterization). One of 6E10-^64^Cu or 6E10-PEG-^64^Cu was injected intravenously into both TgCRND8 and wild type mice and imaged at 10 min, 2 h, 4 h and 12 h. We expected 6E10 to accumulate in the TgCRND8 brain because of enhanced retention from binding to plaques and that PEG-modification would increase the BBB penetration of 6E10. Brain tissue of TgCRND8 mice intravenously injected with 6E10 consistently contained approximately 1% of the injected dose per gram of tissue whereas the brain tissue of wild type control mice contained approximately 0.75% of the injected dose per gram of tissue (Figure 2A). These concentrations remained relatively constant over the 12 h period monitored. Three-factor ANOVA (time, animal, antibody) detected no significant interactions and determined that the animal type was an important factor (F = 22.791, p<0.001): a Holm-Sidak post-hoc test demonstrated significantly more 6E10 antibody in TgCRND8 mouse brains than in wild type at 4 h (p = .041). Concentrations of 6E10-PEG were approximately twice as high in the TgCRND8 mouse brain at 10 min and 1.75 times greater at 2 h when compared to the wild type animal, but decreased from 2% to 1% of injected dose per gram of tissue at 4 h and 12 h (Figure 2B). Three-factor ANOVA demonstrated that the TgCRND8 mouse was an important factor for 6E10-PEG accumulation as well (F = 25.817, p<0.001) and Holm-Sidak post-hoc tests revealed significance at 10 min (p = 0.001) and 2 h (p = 0.009) relative to wild type controls. The concentration of 6E10-PEG was examined in a reference tissue composed of skin and muscle in order to examine temporal changes in concentration. In TgCRND8 mice, the concentration of the 6E10-PEG remained constant in the reference tissue over the 12 h examined whereas the concentration in the brain decreased (Figure 2C). In contrast, in wild type mice, concentrations of 6E10-PEG remained constant over the entire 12 h time frame in both reference and brain tissues (Figure 2D). Thus, 6E10-PEG in the TgCRND8 mouse was the only group that showed changes in concentration in the brain over time, suggesting that antibody-antigen interactions can be used to identify Aβ pathology which differs from other non-specific interactions observed in reference tissues and non-diseased brains in wild type animals.

6E10 concentrations observed in the brain were increased after PEG-modification in both the TgCRND8 mice (Figure 2E) and wild type mice (Figure 2F). Three factor ANOVA confirmed that PEG-modification was a significant factor (F = 29.672, p<0.001) independent of whether the tissue analyzed came from a TgCRND8 (p = 0.003) or wild type mouse (p<0.001). Post-hoc Holm-Sidak tests revealed higher accumulation of 6E10-PEG in TgCRND8 mice when compared to 6E10 at 10 min (p = 0.003) and 2 h (p = 0.002). No significant differences were found between 6E10 and 6E10-PEG in wild type mice. This observation is consistent with previously reported results that PEG-modification prolongs circulation and can enhance permeation across the blood-brain barrier [24]. These results demonstrate that: PEG-modification increases 6E10 brain permeability, 6E10 can be detected using PET and, importantly, there is greater retention of 6E10 in TgCRND8 vs. wild-type mice.

### 3.2 Hyperosmotic Disruption of the Blood-Brain Barrier

**Figure 3:**
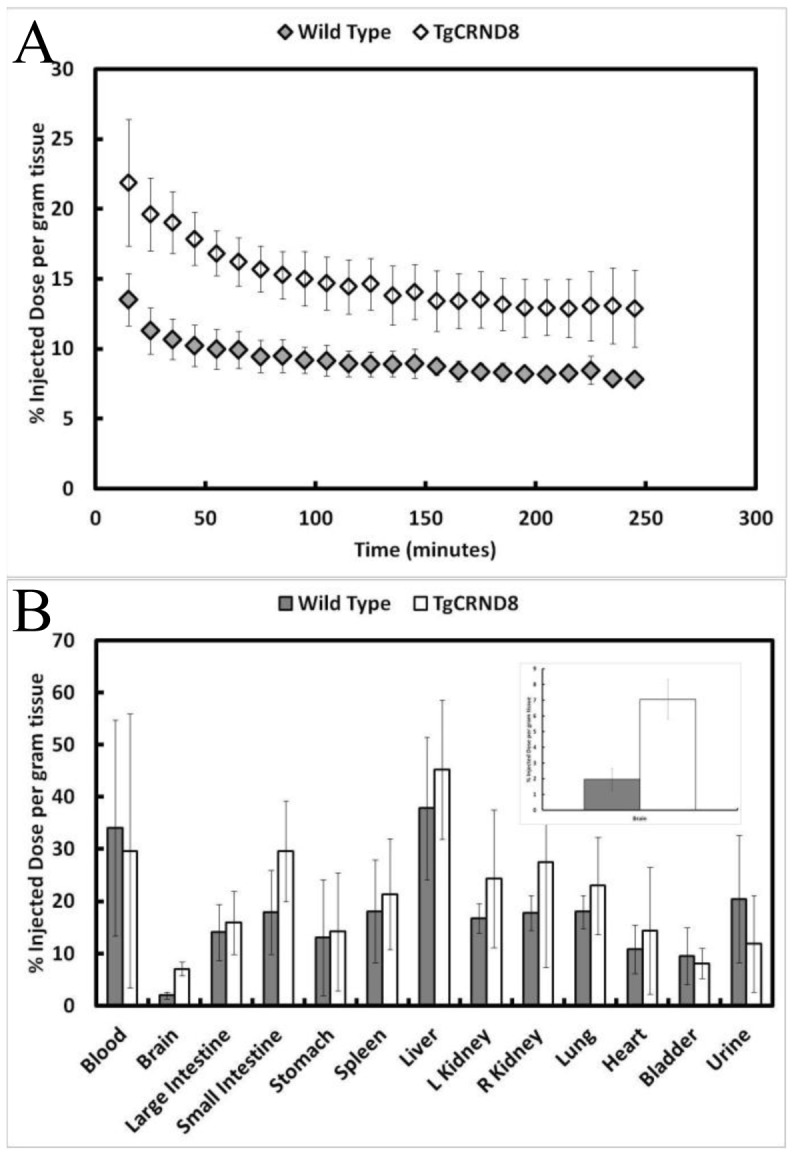
The concentration of 6E10-^64^Cu is higher in the TgCRND8 brain when compared to a wild type control animal after hyperosmotic disruption of the blood-brain barrier. (A) TgCRND8 and wild type mice are dynamically scanned by PET for 4 h after hypersosmotic disruption of the blood-brain barrier and administration of 6E10-^64^Cu. 6E10 levels are consistently higher in the TgCRND8 mouse when compared to the wild type mouse. Two factor ANOVA reveals a significant difference between the TgCRND8 and wild type mice (p<0.001, n = 4, mean ± standard deviation plotted). (B) Post-mortem biodistribution analysis of the organs shows similar distribution of 6E10 in all organs, except the brain. Increased accumulation in the TgCRND8 brain compared to a wild type brain is shown in the inset panel (n = 4, mean ± standard deviation plotted).

The BBB is known to be damaged by the accumulation of Aβ and this could contribute to enhanced accumulation of 6E10 in the extracellular space of the TgCRND8 mouse [25]. We hyperosmotically disrupted the BBB in the TgCRND8 and wild type mice in order to erase any differences in permeability across the two types of mice. Immediately after BBB disruption, the animals were injected with 6E10 and dynamically scanned with a microPET scanner (Figure 3A). Concentrations of 6E10 were roughly twice as high in the TgCRND8 mouse brain (23% of the injected dose per gram of tissue) when compared to the wild type mouse brain (13% of the injected dose per gram of tissue) at the earliest time point. Within 2 h of injection, the concentration of 6E10 in the TgCRND8 and wild type mice reached an equilibrium, where the TgCRND8 levels (15% of the injected dose per gram of tissue) were roughly 50% higher than that in the wild type mouse (10% of the injected dose per gram of tissue). Accumulation of 6E10 in other organs is not significantly different across the TgCRND8 and wild type mice, indicating that the peripheral distribution of 6E10 does not contribute to the differences observed in the brain. Post-mortem analysis of the organs confirms a higher concentration of 6E10 in the TgCRND8 brain tissue in comparison to the wild type tissue (Figure 3B). In the absence of a tight BBB, more 6E10 is observed in the TgCRND8 vs. wild type brain, thereby demonstrating antibody-mediated Aβ imaging is possible in living mice and not merely due to differences in BBB permeability which were minimized through hyperosmotic disruption.

### 3.3 Biodistribution of 6E10-PEG after Intravenous Delivery

**Figure 4:**
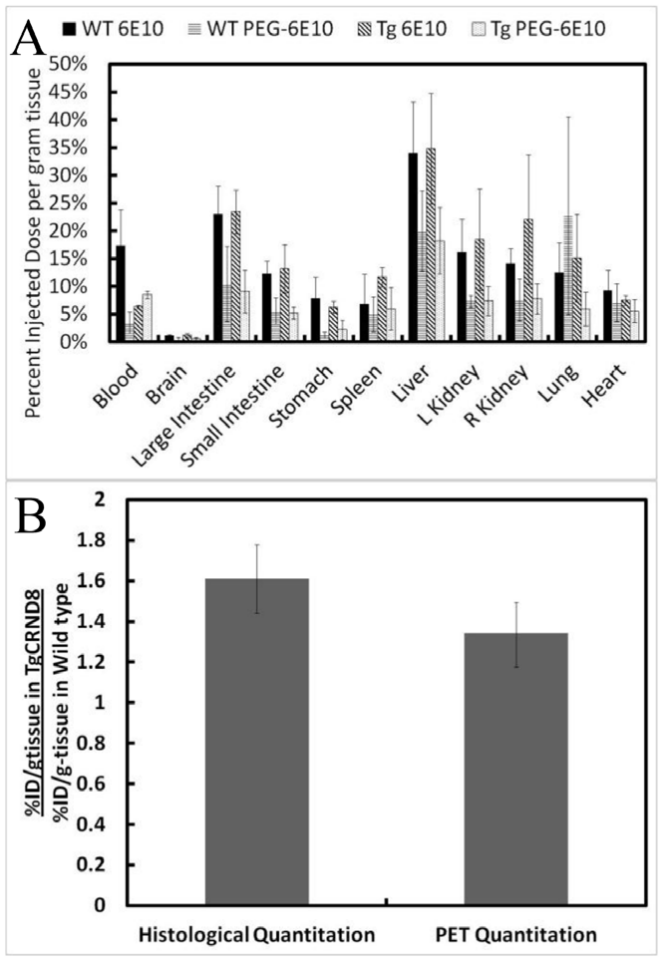
Examination of brain tissue post-mortem confirms higher accumulation of intravenously injected 6E10-^64^Cu in TgCRND8 than wild type mice. (A) Post-mortem biodistribution analysis of all organs 12 h after intravenous injection does not distinguish between TgCRND8 and wild type brain tissue, but does reveal accumulation and removal in liver, kidneys, and gastrointestinal organs (n = 3, mean ± standard deviation plotted). (B) Ratio of % injected dose per gram of tissue TgCRND8 to wild type brain tissue, as measured by gamma counting and PET quantitation, confirms higher accumulation in TgCRND8 than wild type mice 2 h after injection with 6E10-PEG. (n = 3, mean ± standard deviation plotted).

We were interested in the biodistribution of 6E10 and 6E10-PEG after intravenous injection. Post-mortem analysis of organs dissected from TgCRND8 and wild type mice at 12 h after intravenous injection demonstrate that, with the exception of blood, brain, lung and heart, the concentrations of 6E10-PEG were twice as high as that of 6E10 (Figure 4A). Biodistribution is presented as a percentage of the injected dose per gram of tissue to demonstrate accumulation in organs. The total dose detected in animal (% ID/g multiplied by the weight of each organ) approached 100%. In addition to enhancing diffusion across the BBB, PEG-modification is proposed to have a variety of masking effects, potentially mediated through reduced protein adsorption and reduced binding to receptors, which prolongs circulation. Three-factor ANOVA confirmed that PEG-modification was an important factor in biodistribution of 6E10 (F = 65.302, p<0.001). No conclusion could be made about the animal type, which indicates that TgCRND8 mice do not have significantly different biodistributions of 6E10 or 6E10-PEG when compared to wild type mice. A significant interaction between organ and antibody was detected, indicating that PEG-modification has different effects in different organs (F = 3.712, p<0.001). Analysis of brain tissue did not reveal differences between the TgCRND8 and wild type mice, nor was such analysis able to detect differences between PEG-modified and native 6E10. These observations were consistent with quantitation of 6E10 accumulation in brain tissue with PET imaging at 12 h.

In order to confirm that differences observed with PET after intravenous delivery of 6E10-PEG were reliable, we repeated the experiment but sacrificed the animals at 2 h to allow for histological examination of the tissue at a time point where we observed differences in brain tissue accumulation by PET imaging. Brain tissue was dissected and the percent of injected dose per gram of tissue was compared. There was a 1.6 times greater percent of the injected dose per gram of tissue in the TgCRND8 than the wild type mouse, as measured using histological techniques, which is statistically indistinguishable using a t-test (p = 0.110) from the 1.4 times greater accumulation in the TgCRND8 measured with PET imaging (Figure 4B). Thus, histological examination of the brain tissue at 2 h validated PET imaging as a non-invasive technique to measure 6E10-PEG accumulation in brain tissue.

### 3.4 Circulation and Brain Accumulation of IgG-PEG in Mice

**Figure 5:**
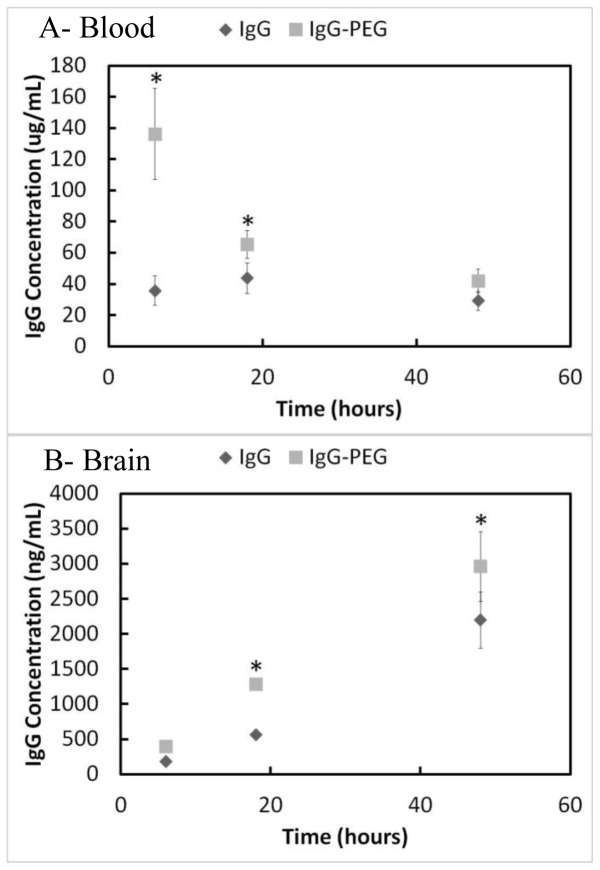
PEG-modification of rabbit IgG results in higher accumulation in brain tissue. (A) PEG-modification of IgG leads to elevated concentrations in the blood at early time points, but is diminished by 48 h. Holm-Sidak post-hoc test after a two-factor ANOVA (antibody and time) revealed significant differences at 6 h (p<0.001) and 18 h (p = 0.027). (B) In contrast, the penetration of IgG into the brain is elevated at the intermediate time points, but by 48 h the effects of PEG-modification begin to diminish in the brain. Holm-Sidak post-hoc test after a two-factor ANOVA (antibody and time) revealed significant differences at 18 h (p<0.001 and 48 h (p<0.001) (n = 4, mean ± standard deviation plotted).

To gain greater insight into effects of PEG-modification on antibody circulation and accumulation, the transport kinetics of IgG vs. IgG-PEG were examined. Rabbit IgG vs. IgG-PEG was injected into 5 month old wild type mice and the blood and brain concentrations of IgG were measured over 48 h at: 6, 18 and 48 h. A two-factor ANOVA (antibody and time) revealed no significant interactions amongst the main effects and revealed that both antibody (F = 37.244, p<0.001) and time (F = 211.672, p<0.001) were significant factors. Blood concentrations of IgG-PEG were significantly elevated when compared to native IgG at 6 and 18 h, but were nearly identical at 48 h (Figure 5A). Brain concentrations of IgG-PEG were elevated at 18 h, but the elevation was reduced at 48 h (Figure 5B). Brain concentrations of IgG-PEG were not directly proportional to blood concentrations across the three time points. At 6 h the concentration of IgG-PEG in the blood was 3.5 fold greater than IgG, however the brain concentration of IgG-PEG only increased 2 fold. At 18 h the concentration of IgG-PEG is 1.5 fold greater than IgG in the blood, yet the concentration of IgG-PEG is 2.25 fold greater than IgG in the brain. If the transport were strictly driven by a concentration gradient in the blood, then brain concentrations would likely be more directly proportional.

## Discussion

Our study demonstrates the use of an anti-Aβ antibody to distinguish Aβ bearing TgCRND8 mice from wild type littermates. TgCRND8 mice can clearly be differentiated from wild type mice using systemically delivered 6E10, with the detection enhanced with 6E10-PEG. PET imaging of brain tissue after intravenous delivery of 6E10 resulted in significantly elevated brain concentrations of 6E10 in the TgCRND8 mice when compared to wild type controls. Brain concentrations of 6E10 remained constant over the 12 h period examined with PET imaging. After 6E10 was conjugated to PEG, we saw increased transport into brain tissue and evidence of Aβ binding.

6E10-PEG concentrations in brain tissue were elevated at 10 min and 2 h, but returned to concentrations observed in wild type controls at 4 h and 12 h, indicating antigen-mediated binding and clearance. In contrast, the concentration of 6E10-PEG in a reference tissue, where no antibody-antigen interactions are expected, remained constant over the entire time frame examined. Further, when the blood-brain barrier was disrupted by infusion with a hyperosmotic sugar solution, and thereby reduced (or eliminated) any differences in vasculature due to Aβ pathology, there is clear accumulation of 6E10 at early time points. Notwithstanding that hyperosmotic disruption of the blood-brain barrier is not a clinical strategy due to the invasive nature of this technique, there is higher accumulation of 6E10 in the brain. Moreover, the clearance profile of 6E10 in the TgCRND8 animal is consistent with the clearance profile observed after intravenous delivery of 6E10-PEG. It is possible that higher uptake of the tracer would be observed in TgCRND8 brain tissue, but the maximum concentration appears to happen before measurements are taken and limits the conclusions that can be drawn. These experiments demonstrate that intravenous delivery of 6E10-PEG can provide a non-invasive assessment of Aβ pathology in living animals.

These results advance the development of antibody-based contrast agents in Alzheimer's disease by demonstrating differentiation of mice bearing Aβ pathology from wild type littermate controls using a systemically delivered contrast agent and non-invasive live-animal medical imaging techniques. These results are consistent with the impressive binding to Aβ plaques using gadolinium labelled antibodies reported by Ramakrishnan et al [15]. More recently, Koffie et al used an antibody-coated nanocarrier to label plaques, but also used histological techniques to quantify the signal [17]. Koffie et al made an important advance by showing non-invasive detection of nanoparticle accumulation in wild type brain tissue. Herein, we build on that discovery by non-invasively detecting pathology without having to use histological techniques that were instrumental in previous studies.

Importantly, it is well-established that if 6E10 is able to gain access to the central nervous system it will bind to plaques. This was substantiated in our previous study where 6E10 labelled plaques were demonstrated in TgCRND8 mice after cortical injection [20]. Increased accumulation of 6E10 in the TgCRND8 mice was observed with two delivery routes (intravenous and intracarotid) and two different formulations of 6E10 (native and PEG-modified). For these reasons, the increased accumulation of 6E10 in the brain of TgCRND8 mice is a reasonable proxy for Aβ pathology detection.

Conjugation of PEG to macromolecules is known to prolong circulation through reduced protein adsorption [24] and reduced binding to receptors that may lead to clearance [26]. Prolonged circulation results in greater serum concentrations that drive greater accumulation of an antibody in low permeability organs such as the brain. The higher concentrations of 6E10-PEG in brain tissue observed with PET in this study are likely partly driven through this process. Since it is unlikely that PEG would provide an enhanced protective effect in the TgCRND8 animals over wild type controls, enhanced accumulation of 6E10-PEG in the TgCRND8 brain is attributed to retention after binding to Aβ plaques.

In addition to the passive transport of 6E10-PEG driven through increased serum levels of antibody, PEG can also bind to LDL-receptors on the BBB and promote active transport of macromolecules. PEG is proposed to work through two pathways: selective adsorption of lipoproteins and direct binding to LDL-receptors [18]. While LDL receptors are not upregulated in Alzheimer's disease [27], the enhanced accumulation of 6E10-PEG in the TgCRND8 mouse brain over wild type controls at early time points is likely due to greater binding to Aβ plaques.

A potential source of signal in the brain tissue of wild type and transgenic animals is antibody circulating in the brain vasculature that has not penetrated into the brain tissue. In order to quantify the contribution of the brain vasculature, we estimated the amount of blood in the brain tissue and compared this to the signal measured with PET imaging. The typical blood volume of a mouse is estimated at 1.5 mL while the typical mass of the mouse brain is approximately 400 mg with an estimated 0.01 mL of blood circulating in 1 gram of brain tissue [28]. If 100% of the dose were in the blood, this would correspond to a measurement of 0.67% of the injected dose per gram of brain tissue, which is considerably lower than PET measurements made in all animals. Further, it is unlikely that 100% of the dose remains in circulation at any of the time points measured because antibodies are known to rapidly permeate into a variety of organs [29]. Therefore, we are able to conclude that the signal measured is not due to the plasma pool.

Anti-Aβ antibodies have been widely studied as therapeutics to remove Aβ accumulation in the brain through passive or active immunization [30]. Thus 6E10-PEG has the potential to be utilized as both a therapeutic and diagnostic agent. Antibodies can remove Aβ plaques from the brain through a variety of chemical and biological processes. While the effect of 6E10-PEG on the plaque load in the TgCRND8 mice was not quantified in this study, this is an interesting question for further examination as removing Aβ from the brain can alter brain vasculature and impact biocompatibility [31].

Copper-64 was chosen as a model radionuclide for this study primarily because its relatively long half-life of 12 h allowed the PET measurements to be made at this time point. In this study earlier time points (10 min and 2 h) were more useful in differentiating between wild type and transgenic animals and therefore demonstrate that an isotope with a shorter half-life could also be utilized. While the dosages of Copper-64 used in this study are already low, the selection of a different isotope could further improve biocompatibility.

This study provides a platform for further research with antibodies that have specificity to Aβ isoforms and are pathologically relevant, such as Aβ oligomers, or earlier stage Aβ plaques. In previous studies, 6E10 was shown to bind to plaques in 8 month old TgCRND8 mice [20], which have late-stage plaques yet unclear pathological relevance. The imaging methods described herein will allow relevant antibodies to be studied and thereby provide both a greater understanding of Aβ pathology and a diagnostic and monitoring tool.

## Conclusion

Chemical modification of the classical anti-Aβ antibody, 6E10, with polyethylene glycol increases transport across the BBB in the TgCRND8 mouse model of Alzheimer's disease. 6E10-PEG conjugates can be used to image Aβ pathology using live-animal PET and differentiate animals with Aβ pathology from wild type control animals. This study demonstrates antibody-mediated non-invasive imaging of Aβ pathology and enables the development of highly specific Aβ imaging tools.

## Supporting Information

Figure S1 (TIF)

Supplementary Information S1 (DOCX)

Table S1 (TIF)
