# Supplementary figures and images for: Anti-Amyloid-β-Mediated Positron Emission Tomography Imaging in Alzheimer's Disease Mouse Brains
Source: PLoS One. 2012 Dec 21;7(12):e51958. doi: 10.1371/journal.pone.0051958 (PMC3528731; doi:10.1371/journal.pone.0051958)

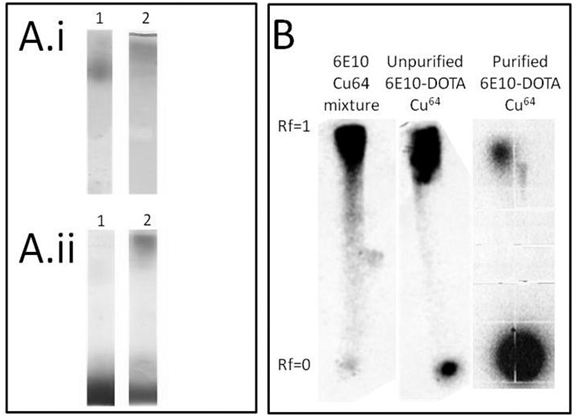

Supplement: Figure S1 — (TIF) [file pone.0051958.s001.tif]

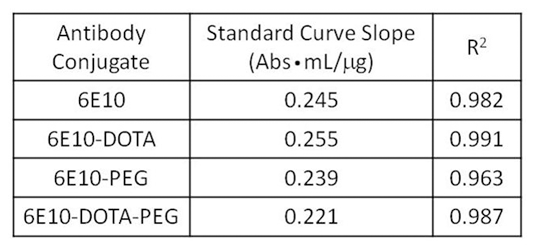

Supplement: Table S1 — (TIF) [file pone.0051958.s003.tif]
