# Supplementary material for: Anti-Amyloid-β-Mediated Positron Emission Tomography Imaging in Alzheimer's Disease Mouse Brains
Source: PLoS One. 2012 Dec 21;7(12):e51958. doi: 10.1371/journal.pone.0051958 (PMC3528731; doi:10.1371/journal.pone.0051958)
Supplement: Supplementary Information S1 — (DOCX) [file pone.0051958.s002.docx]

**Supplementary Information**

**Supplementary Materials and Methods S1**

Primary amines on the classical anti-amyloid-β antibody 6E10 (SIG-39320, Covance Research Products Ltd) were modified with either or both the chelating macrocyclic 1,4,7,10-Tetraazacyclododecane-1,4,7,10-tetraacetic acid mono (N-hydroxysuccinimide ester), DOTA-NHS (B-280, Macrocyclics Inc.)) and a 10 kDa polyethylene glycol mono (N-hydroxysuccinimide ester) chain (Rapp Polymere) through NHS coupling chemistry. 6E10 was diluted in PBS to a concentration of 500 mg/mL and kept at room temperature. 10 times molar excess of 1 mg/mL DOTA-NHS in PBS or 1 mg/mL PEG-NHS in PBS or both 1 mg/mL DOTA-NHS and 1 mg/mL PEG-NHS in PBS was added to the 6E10 solution and shaken at room temperature for 4 h to allow the reaction to proceed. Excess DOTA-NHS was purified from 6E10-DOTA conjugates by centrifugal filtration using an Amicon Ultra-0.5mL centrifugal 30 kDa MWCO filter unit (UFC5030, Millipore). Excess PEG-NHS was purified from 6E10-PEG conjugates by centrifugal filtration using an Amicon Ultra-0.5mL centrifugal 100 kDa MWCO filter unit (UFC5100, Millipore). Mixtures were washed 4 times with PBS. Modification of 6E10 with PEG was examined with sodium dodecyl sulfate polyacrylamide gel electrophoresis (SDS-PAGE). A non-reducing 5% acrylamide gel was resolved for 1 h at 100 V. Protein was visualized with SimplyBlue SafeStain (Invitrogen) and PEG was visualized using a barium iodine stain previously described [[32](#_ENREF_32)]. 6E10 binding to amyloid β was measured after chemical modification with an in-house direct ELISA. Plates were coated overnight at 4 ºC with 10 mg/mL amyloid-β(1-42) (Sigma-Aldrich) peptide in 0.1 M ammonium hydroxide. A standard curve of 6E10 ranging from 1 ng/mL to 1 mg/mL was prepared along with identical dilutions of each 6E10 conjugate. 6E10 standards and samples were incubated on the plates for 2 h, followed by incubation with a goat anti-mouse alkaline phosphatase detection antibody (Sigma-Aldrich) and developed with a pNPP solution (Sigma-Aldrich).

With the goal of improving BBB permeability, 6E10 was modified with 10 kDa PEG chains, which was verified using SDS-PAGE with a Coomassie-blue protein (Supplementary Figure 1Ai) stain and bromide iodine PEG stain (Supplementary Figure 1Aii), both of which overlapped. To ensure chemical reaction, a control was conducted: when a mixture of inactivated PEG-NHS was incubated overnight with 6E10, the PEG and 6E10 signals did not overlap indicating the PEG and 6E10 limited to no non-covalent interaction. Thus, successful modification of 6E10 with PEG occurred only with active PEG-NHS.

In order to immobilize ^64^Cu onto 6E10 in a short period of time, 6E10 was covalently linked to a copper chelator, DOTA, using similar NHS chemistry. Immobilization of DOTA-NHS to 6E10 was confirmed by performing instant thin-layer chromatography (ITLC) on radiolabelled 6E10 conjugates. Supplementary Figure B.i shows ITLC of a mixture of 6E10 and ^64^Cu, B.ii shows ITLC on a mixture of 6E10-DOTA and ^64^Cu and B.iii shows purified 6E10-DOTA-^64^Cu. These ITLC images demonstrate that 6E10 cannot chelate ^64^Cu without DOTA and that high purity 6E10-^64^Cu can be prepared for injection. Standard curves from a direct ELISA of 6E10, 6E10-DOTA, 6E10-PEG, and 6E10-PEG-DOTA show the slopes of the standard curves are affected by less than 10%, indicating that the modified 6E10 retains high antigen binding capabilities (Supplementary Table 1). Successful modification of 6E10 with PEG and DOTA allows for testing of improved BBB penetration with PET imaging.

**Legends to Supplementary Figures and Tables**

**Supplementary Figure S1:**

**Anti-amyloid-β antibody, 6E10, is modified with a 10 kDa Polyethylene glycol chain and a heavy metal chelating moiety, 1,4,7,10-Tetraazacyclododecane-1,4,7-tris(t-butyl acetate)-10-acetate-mono-N-hydroxysuccinimide-ester) (DOTA-NHS).**

(A) SDS-PAGE of 6E10 and 6E10-PEG demonstrate immobilization of PEG chain to 6E10 molecule. Lane 1 is a mixture of 6E10 and PEG, lane 2 is 6E10-PEG conjugates. Proteins stained with simplyBLUE stain (A.i) and PEG chains stained with Iodine stain (A.ii). (B) Instant thin-layer chromatography separates ^64^Cu immobilized on antibody from free ^64^Cu and is viewed with phosphor screen. The origin is labelled with a retention factor (Rf) of 0 and the solvent front with a retention factor of 1. Lane 1 is free ^64^Cu (Rf=1) and 6E10, lane 2 is mixture of ^64^Cu (Rf=1) and 6E10-DOTA (Rf=0) and lane 3 is purified 6E10-DOTA-^64^Cu (Rf=0) with less than 5% free ^64^Cu (Rf=1).

**Supplementary Table S1: Slopes of standard curves of direct ELISA of 6E10 Conjugates.** The slope of the standard curve of 6E10 changes by less than 10% after modification with DOTA and/or PEG, as quantified by a direct amyloid-β ELISA, thereby demonstrating that 6E10 remains active after modification with polyethylene glycol and DOTA.
